# Integrated genomic and transcriptomic profiling reveals distinct molecular features of colorectal cancer in an Indian cohort

**DOI:** 10.1016/j.neo.2026.101381

**Published:** 2026-10-16

**Authors:** Anil Vashapaka, Ravikanth Vishnubhotla, Rao GV, Pradeep Rebala, Sanjeev M Patil, Mahesh Shetty, K Srinivas Naik, Anuradha Sekaran, Nageshwar Reddy D

**Affiliations:** a Asian Healthcare Foundation, Plot No 2/3/4/5, Survey No 136/1, Mindspace Road, Gachibowli, Hyderabad, Telangana, 500032, India; b AIG Hospitals, Plot No 2/3/4/5, Survey No 136/1, Mindspace Road, Gachibowli, Hyderabad, Telangana, 500032, India; c Centre for Plant Molecular Biology, Osmania University, Tarnaka, Hyderabad, Telangana, 500007, India

**Keywords:** Colorectal Cancer, Whole-exome sequencing, Transcriptome profiling, Genomic landscape, Somatic variants

## Abstract

Colorectal cancer (CRC) exhibits substantial molecular heterogeneity, requiring integrative approaches to define clinically meaningful tumour subtypes. Although large international consortia have extensively profiled CRC, global datasets underrepresent many ethnicities. Understanding how local tumors align with/diverge from globally characterized cohorts is essential to contextualize disease biology and refine precision oncology strategies. This study was designed as a comparative, hypothesis-generating analysis benchmarked against a Global Genomic Landscape (GGL) of 2,023 CRC tumors.

Whole exome and transcriptome sequencing was performed on pathologically confirmed tumors (N = 20) and normal tissues (N = 5) using the Illumina HiSeqX. Bioinformatic pipelines identified somatic variants and dysregulated genes, which were compared with GGL data.

We identified 814 dysregulated genes and ∼16,440 somatic variants. While the single base substitution (SBS) profile was similar to GGL, one double base substitution signature (DBS17) and two insertion–deletion signatures (ID7, ID19) were unique to our cohort. Six driver genes (ACVR2A/BAX/PRDM2/RGS12/RNF43/TCF7L2) with recurrent variants were observed in our cohort. Only three genes with emerging roles in CRC (*MET/SOX9/TGIF1*) overlapped across dysregulation, somatic variation, and GGL datasets. Indel hotspot variants in *EIF2B3* (p.Ala151GlnfsTer57) and *UBR5* (p.Glu2121LysfsTer28) were exclusive in non-survivors. The prevalence of canonical Western driver mutations (*APC, TP53, KRAS, PIK3CA, SMAD4*) was markedly lower in our cohort. Few drug-targetable genes overlapped with GGL; however, several approved or high-confidence targets were identified.

Compared with the global genomic landscape, our findings suggest potential population-associated differences in driver architecture and therapeutic opportunities that warrant validation in larger independent Indian cohorts.

## Highlights

• Unique DBS17, ID7, and ID19 mutational signatures found in this colorectal cohort.
• Prevalence of canonical Western drivers (APC, TP53, KRAS) was markedly lower.
• Indel hotspot variants in EIF2B3 and UBR5 were enriched in non-survivors.
• MET, SOX9, and TGIF1 overlapped across variant and global genomic datasets.

## Graphical abstract

**Figure:**
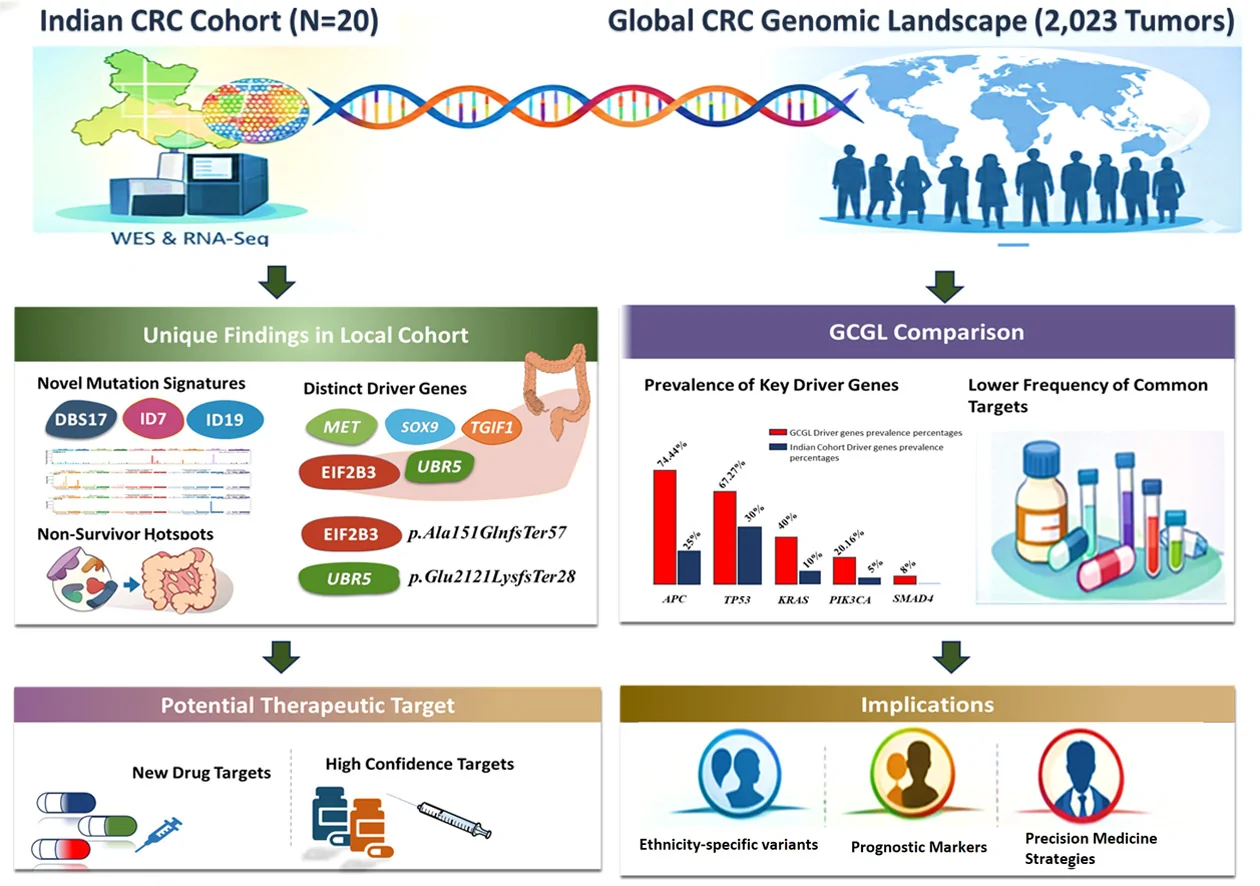

## Introduction

Colorectal cancer (CRC) is among the most common malignancies worldwide, ranking third in incidence and second in cancer-related mortality [1]. In 2020, CRC accounted for approximately 1.93 million new cases and over 935,000 deaths globally [2]. While the highest burden is observed in developed regions (Europe, North America, and Australia), low- and middle-income countries are experiencing a rapid increase in CRC incidence, driven by shifts in lifestyle, dietary habits and aging populations [[3], [4], [5]]. In India, CRC remains less prevalent than in Western countries, but its incidence is rising steadily [6]. It is now among the top five cancers in urban areas, with an estimated 25,000-30,000 new cases annually, particularly in southern and northeastern regions. Notably, CRC in India is often diagnosed at a younger age and at more advanced stages, resulting in poorer outcomes and lower five-year survival rates [7,8]. These epidemiological trends underscore an urgent need for molecular-level studies of colorectal tumors in the Indian population.

A recent large-scale study [9] analysed the genomic landscape of ∼2,000 colorectal cancer tumors, identifying a broad array of mutations including single nucleotide variants (SNVs), structural variants (SVs), and copy number alterations (CNAs). This comprehensive analysis expanded the list of genes implicated as potential CRC drivers to over 250, highlighting the complexity of the disease's genetic underpinnings.

Most of the global datasets do not represent the ethnic variation found across the world [10]. Generation and comparison of multi-omic data in ethnicities not represented in these large datasets offers substantial advantages. Larger studies typically benefit from enhanced statistical power and greater precision in estimating effect sizes, making their results more reliable and generalizable to broader populations [11]. When the findings from a smaller dataset align with those from a larger study, it lends additional credibility and external validity to the conclusions, even if the initial sample was limited [12]. This comparative approach also allows researchers to contextualize their findings within the existing body of literature, identify consistent trends, and generate hypotheses for further exploration. We generated whole exome and transcriptome sequencing data in the same cohort of tumours with CRC and compared the data with a recently published global genomic landscape [9]. We identified similarities with the global landscape, and most importantly highlight several findings observed only in our cohort of tumours.

## Materials and Methods

A total of 20 CRC tumors and 5 control colorectal tissues were collected in RNAlater for this prospective study conducted between May 2013 and December 2017. All patients were followed for a minimum of 36 months. Pathologically confirmed malignant colorectal tumors were obtained from patients undergoing surgery, while control tissues were collected from patients undergoing surgery for non-malignant conditions. Patients who had received neoadjuvant chemotherapy, had diffuse peritoneal metastasis at initial surgery, or had undergone only palliative procedures (such as diversion stoma) were excluded. DNA was isolated using the Qiagen kit according to the manufacturer's protocol. Whole exome sequencing libraries were prepared (Twist Human Customized Core exome kit) and sequenced on the Illumina Hiseq platform to generate 2 × 150 bp reads per sample with a minimum depth of 500 × to identify somatic variants. Whole transcriptome sequencing data were generated and analysed as described previously [13]. To identify de novo mutational signatures, SigProfilerExtractor was run independently using the analysis-ready Mutect2 VCF files before application of the downstream in-house prioritization criteria, where Non-negative Matrix Factorization (NMF) was performed on Single-Base Substitution (SBS), Double-Base Substitution (DBS), and Small Insertion and Deletion (ID) data [14]. Subsequently, these de novo patterns were decomposed into known reference signatures using the COSMIC repertoire (v3) to determine the activity and contribution of established mutational processes [15]. Pairwise associations between mutational signatures were assessed, and signatures were compared between survivors and non-survivors, in addition to the anatomical location. Dysregulated genes from transcriptome data and genes with somatic variants from our cohort were compared to annotated driver genes from the global landscape. Genes with recurrent mutations and driver genes with secondary hits were identified. Copy number alterations, Focal copy number gains and deletions were identified using CNVKit and GISTIC [16,17]. These CNA alterations were compared between groups to identify similarities and unique genomic alterations. Transcriptome and whole exome sequencing data from our cohort were also compared to genetic features with approved therapies reported in the global landscape, and gene targets with approved and high-confidence drug targets were identified. The study was approved by the Institutional Research and Ethics Committee of the Asian Institute of Gastroenterology (AIG/IEC34/07.2019-19), and written informed consent was obtained from all participants. The study protocol adhered to the ethical guidelines of the 1975 Declaration of Helsinki. A detailed methodology is provided in the Supplementary Information. (Detailed methodology given in supplementary document)

## Results

### Clinical characteristics of the tumours

The clinical and demographic characteristics of our cohort are given in supplementary Table 31 and previously described by Avanthi et al. [18].

### Transcriptome sequencing

We identified 814 significantly dysregulated genes of which 569 were upregulated and 245 were downregulated from a total of 4189 genes that were dysregulated across the tumours. The significant genes were compared against the global landscape.

### Whole exome sequencing

WES identified 16,440 somatic variants across 20 tumors through our pipeline (Supplementary Figure 1a) for subsequent comparisons with the global landscape. A detailed methodology is given in the supplementary methods.

## Mutational Signatures

The mutational-signature analysis was performed independently using the analysis-ready VCF files generated by the Mutect2 workflow. The mutational-signature analysis identified 45,943 single-base substitutions (SBS), 875 double-base substitutions (DBS), and 6,702 insertions/deletions (ID), giving a combined total of 53,520 mutation records across these three mutation classes.

### Single base substitution (SBS)

Non-negative Matrix Factorization (NMF) of SBS data revealed three *de novo* signatures: SBS96A, SBS96B, and SBS96C. SBS96A (44.6% of mutations) that featured C > T substitutions at GpC contexts, SBS96B (27.4%) showed dominant C > T mutations and SBS96C (27.9%) exhibited a localized T [C > A] T peak, indicative of a *POLE* exonuclease domain mutation. Mapping these to COSMIC v3 confirmed no novel signatures and they resembled established composites (e.g., SBS96C matched SBS1, 5, 10a, and 10b with 0.98 cosine similarity). Notably, one tumour emerged as an ultra-hypermutator, contributing 12,373 variants, or 27.0% of the entire cohort's total variant burden (Fig. 1a). This tumour was dominated by SBS10b (Deficient *POLE* Proofreading) that accounted for 5,504 variants (44.5% of its total).

**Fig. 1:**
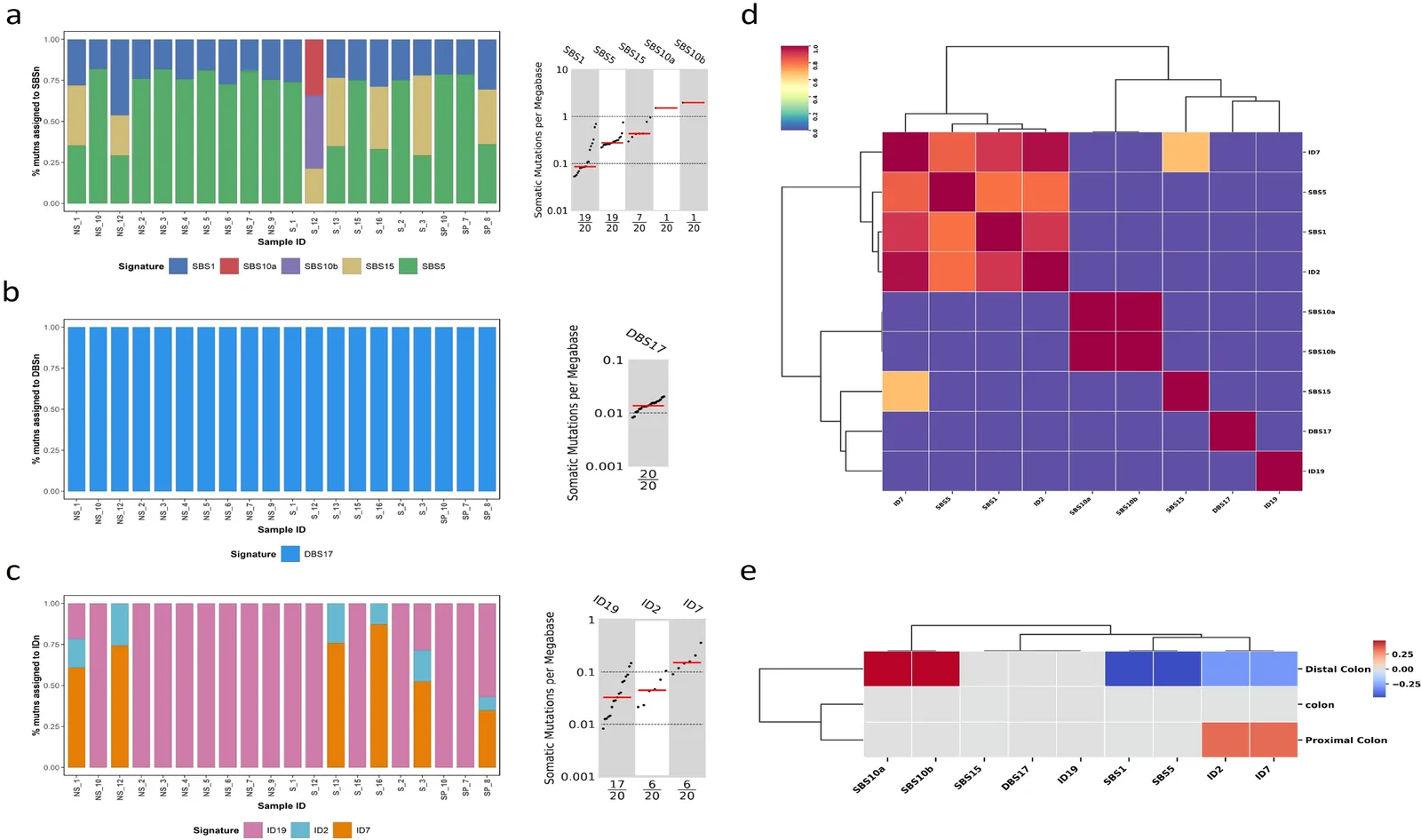
Mutational Signature contributions per sample and the mutation burden in the tumours. A) SBS (Single base substitution) mutation signatures activity plot across the samples and the TMB across the SBS types. A high TMB (tumour mutation burden) is seen in SBS10a and SBS10b. B) DBS (double base substitution) mutational signature (DBS 17) activity across the tumours and the TMB of DBS across the tumours. The presence of DBS17 signature was exclusive to our cohort. C) ID (Insertion deletion) mutational signature activity across the tumours and the TMB across the ID types. Highest TMB was noted in ID7 and ID19 had TMB present in almost all samples observed our cohort (17/20). D) Pairwise associations between Mutational Signatures (Bonferroni p < 0.05). E) Mutational signatures and association with anatomical locations.

### COSMIC decomposition reveals five active signatures

The 45,943 substitutions across all tumors were decomposed into five COSMIC signatures: SBS1, SBS5, SBS10a, SBS10b, and SBS15. Signature details are summarized in Table 1.

**Table 1:** The frequencies and activities of each signature (SBS, DBS and ID) in our cohort.

Type	ID	Proven or suggested cause	Frequency in Global CRC Data	Frequency in this study	Mean activity in this study	Variance (activity) in this study	COSMIC v3 database frequency in CRC	No. of Survivors	No. of Non-Survivors
SBS	1	Clock	99.00%	95%	16.44%	10.25%	98.22%	9	10
SBS	5	Clock	99.26%	95%	31.12%	22.77%	87.99%	9	10
SBS	10a	POLE	1.83%	5%	7.94%	19.61%	2.85%	1	0
SBS	10b	POLE	0.84%	5%	10.37%	32.89%	4.16%	1	0
SBS	15	MMRd	6.77%	35%	19.70%	11.88%	7.97%	5	2
DBS	17	Unknown	Not found	100%	1.48%	0.00%	NA	10	10
ID	2	Replication slippage	99.95%	30%	1.69%	0.10%	64.71%	4	2
ID	7	MMRd	Not found	30%	5.89%	2.05%	29.41%	4	2
ID	19	Unknown	Not found	85%	5.42%	0.46%	NA	8	9

CRC: Colorectal Cancer; v3: version 3; SBS: Single base substitution; DBS: double base substitution; ID: Insertion deletion; POLE: DNA Polymerase Epsilon, Catalytic Subunit; MMRd: Mismatch repaire deficient; %: percentage; NA: Not applicable.

### Double base substitution (DBS)

De novo extraction revealed a single signature, DBS78A. Mapping this to the COSMIC catalogue identified DBS17 (0.96 cosine similarity) that explained 100% of cohort activity.

DB17 that was observed in our cohort was also reported in COSMIC, but was not detected in the Global CRC Data comparator. No other novel signatures were identified. DBS17 features tandem variants—notably GC>NN (AA), TC>NN (AA), and TG>NN (CA)—with unknown etiology. The total DBS burden was low (875), distributed across all tumors without hypermutator outliers (Fig. 1b).

### Insertions and Deletions (ID) mutational signature

De novo extraction identified two signatures: ID83A and ID83B. COSMIC mapping resolved these into three established signatures (Fig. 1c): ID83A (0.91 similarity) was a composite of ID2 (22.12%) and ID7 (77.88%), while ID83B (0.92 similarity) matched ID19 (100%). Together, these signatures fully explained the cohort’s 6,702 insertions and deletions (Fig. 1c). DNA mismatch repair (MMR) deficiency (ID2 and ID7) dominated the landscape; ID19’s etiology remains unknown (Table 1). Notably, ID7 and ID19 appear only in our cohort and are unreported in previous global landscapes for this tumor type.

### Pairwise Associations Between Mutational Signatures

ID7 associated strongly with SBS1, SBS5, and ID2 (Fig. 1d). SBS5 also correlated positively with SBS1 and ID2, while DBS17 associated with ID19 (Fig. 1d). Expectedly, the POLE-linked sub-signatures SBS10a and SBS10b demonstrated a moderate positive association. In contrast, we found strong mutual exclusivity between two distinct clusters: the ID7, SBS5, SBS1, and ID2 group and the SBS10a, SBS10b, SBS15, DBS17, and ID19 group (Supplementary Table 1).

### Mutational Signatures between Survivors and Non-survivors

MMR-deficient signatures (SBS15, ID7) occurred more frequently in survivors than non-survivors (50% vs. 20%). Similarly, ID2 (replication slippage) appeared more in survivors than non-survivors (40% vs. 20%). In contrast, SBS1 and SBS5 appeared near-uniformly across all tumors, regardless of clinical outcome. The POLE hypermutation signatures (SBS10a and SBS10b) occurred in only one survivor, while DBS17 and ID19 were distributed equally between groups.

### Mutational Signatures and anatomical locations

Indel signatures (ID2/ID7) associated with proximal colonic tumors, while the single POLE case (SBS10a/b) was distal (Fig. 1e; Supplementary Table 2).

### Annotation of SNV drivers

The global landscape classified 193 genes as drivers. Comparing our whole exome data against this list identified 133 driver genes harbouring somatic variants (Supplementary Table 3; Fig. 2, Supplementary Figure 2). Of these, 11 genes exhibited recurrent mutations (Table 2). Intersecting our dysregulated transcriptome (N = 814) with the 193 drivers revealed only three genes: *MET, SOX9*, and *TGIF1*.

**Fig. 2:**
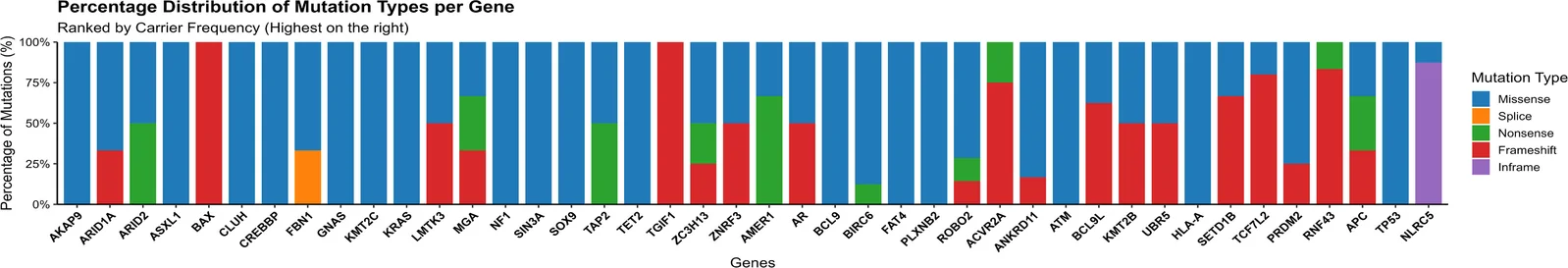
Mutation type and the proportion across 133 driver genes in our cohort of tumours.

**Table 2:** Recurrent Driver mutations.

Gene	Mutation	Carrier	Non-Carrier	Frequency	Non-Survival	Survival
*KRAS*	chr12_25245350_C_T_p.Gly12Asp	1	19	0.05	0	S1
*UBR5*	chr8_102277120_CT_C_p.Glu2121LysfsTer28	1	19	0.05	NS12	0
*SETD1B*	chr12_121804751_AC_A_p.His8ThrfsTer27	2	18	0.10	0	S3,S13
*KRAS*	chr12_25245350_C_A_p.Gly12Val	1	19	0.05	NS4	0
*EIF2B3*	chr1_44941509_CT_C_p.Ala151GlnfsTer57	2	18	0.1	NS1,NS12	0
*BCL9L*	chr11_118899942_TG_T_p.Pro1127HisfsTer21	1	19	0.05	0	S3
***ACVR2A***	Chr2_148683686_A_-_p.Lys329ArgfsTer5	3	17	0.15	NS1	S3,S13
***BAX***	Chr19_49458971_G_-_p.Glu4ArgfsTer19	2	18	0.1	NS12	S3
***PRDM2***	Chr1_14108749_A_-_p.Val1289PhefsTer74	2	18	0.1	NS12	S13
***RGS12***	Chr4_3432431_C_-_p.Gln644ArgfsTer49	2	18	0.1	NS12	S13
***RNF43***	Chr17_56435161_C_-_p.Gly532ValfsTer41	4	16	0.2	NS1,NS12	S3,SP8
***TCF7L2***	Chr10_114925317_A_-_p.Ser387ValfsTer8	3	17	0.15	NS1	S3,SP8

(Bold gene names indicate that they match the driver genes reported in the global landscape, however, they have different recurrent mutations identified in our cohort).

### Enrichment of driver variants based on survivor status

While no driver variants reached statistical significance between groups, several trends emerged. Non-survivors showed a higher frequency of *APC* and *PRDM2* variants (40% vs. 10%), whereas *TP53* and *PDE4DIP* variants were more common in survivors (40% vs. 10%; Supplementary Table 4). Most other genes displayed similar frequencies across both groups, likely representing ubiquitous CRC drivers.

### Frequency of recurrent driver mutations

Global data identify recurrent driver mutations in 59 genes, and data from our cohort matched 11 of these (Table 2). Specifically, two of these genes harboured recurrent variants identical to the global dataset (Supplementary Table 5). Conversely, we identified six other driver genes with recurrent variants not previously reported globally. Supplementary Table 6 summarizes the 48 recurrent variants identified, 17 were exclusive to non-survivors and three to survivors.

### Recurrent mutations in our Cohort

Among 9,120 genes with somatic variants, we identified 435 with recurrent mutations (at least two occurrences). Of these, 48 genes harboured pathogenic or damaging variants (PolyPhen2 and SIFT), with primary functions in immune evasion or as tumour suppressors (Supplementary Table 6).

### Distinctive Driver Gene Distribution

Our cohort’s driver distribution significantly diverged from global CRC patterns (Supplementary Table 7). *NLRC5* mutations dominated (70% prevalence), followed by *TP53* (30%), *APC* (25%), *TCF7L2* (25%), and *RNF43* (25%). *ACVR2A* showed moderate activity (20%), while *KRAS, ARID1A, PIK3CA*, and *FBXW7* were infrequent (≤ 10%).

### Indel hotspots in driver genes

The global landscape identified nine indel hotspots across eight genes. Our cohort matched with two genes exactly: *EIF2B3* (p.Ala151GlnfsTer57) and *UBR5* (p.Glu2121LysfsTer28). While four other genes matched the global list but differed in indel type and position (Supplementary Table 8). Beyond the 133 somatic driver genes, we identified 38 additional indels in 25 genes, including 12 hotspot mutations shared by eight drivers (Supplementary Table 9). Notably, *ACVR2A* p.Lys329ArgfsTer5 occurred in 15% of the cohort, while other indels appeared in single tumors. Other recurrent indels and the top 10 affected genes with identified indels are summarized in supplementary Table 10.

### Indel hotspots and association with survival status

Two somatic indels, *EIF2B3* p.Ala151GlnfsTer57 and *UBR5* p.Glu2121LysfsTer28, were exclusive to non-survivors and matched the variants reported in the global data. This descriptive pattern was unadjusted for nodal stage and should not be interpreted as a causal driver of tumor progression. Details of other indels and their association with survival status are summarized in supplementary Tables 8, 9,10.

### Status of pathogenic variants in driver genes

Of 131 global landscape driver genes, our cohort shared 15 genes harbouring 26 pathogenic variants. These variants were distributed with a median of 1.5 per gene (IQR: 1.0–3.75; Supplementary Figure 3a, Supplementary Table 11). This narrow range indicates most driver genes in our cohort share few pathogenic variants.

### Status of pathogenic variants in other genes

We identified 175 cohort-specific pathogenic variants across 159 genes. This distribution is heavily skewed (median: 2; IQR: 1–18), indicating a small fraction of genes harbours a disproportionately high variant burden (Supplementary Figure 3b, Supplementary Table 12).

Beyond these, we identified 4,952 variants of uncertain significance (VUS) predicted as damaging by SIFT and PolyPhen2, spanning 3,840 genes. These VUS clustered tightly (median: 81; IQR: 72–125 per gene; Supplementary Figure 3c, Supplementary Table 13). This high density suggests these genes are either notably large or highly intolerant to variation, leading to the accumulation of numerous predicted damaging VUS.

### Comparative Analysis of Driver Genes Between the Global Landscape and Our Cohort

To contextualize our cohort, we compared four datasets: Global CRC Landscape Drivers (GCLD), our Whole Exome Data (WED; 9,120 genes), our Transcriptomic Data (TD; 814) and Previously reported CRC Drivers (PD; 973 genes). The PD set represents the accumulation of all candidate drivers from earlier studies (Supplementary Table 14).

The WED set (9,120 genes) was the largest, reflecting the broad somatic variants. We found significant overlap between PD and WED (531 genes; 54%). However, a more stringent analysis identified 45 high-confidence somatic drivers (4.6%) supported by both prior evidence and our specific samples (Supplementary Table 15). Within this central subset, only two genes *SOX9* and *TGIF1* were present across all datasets (Supplementary Table 16, 17), confirming the stringent intersection observed in Fig. 3.

**Fig. 3:**
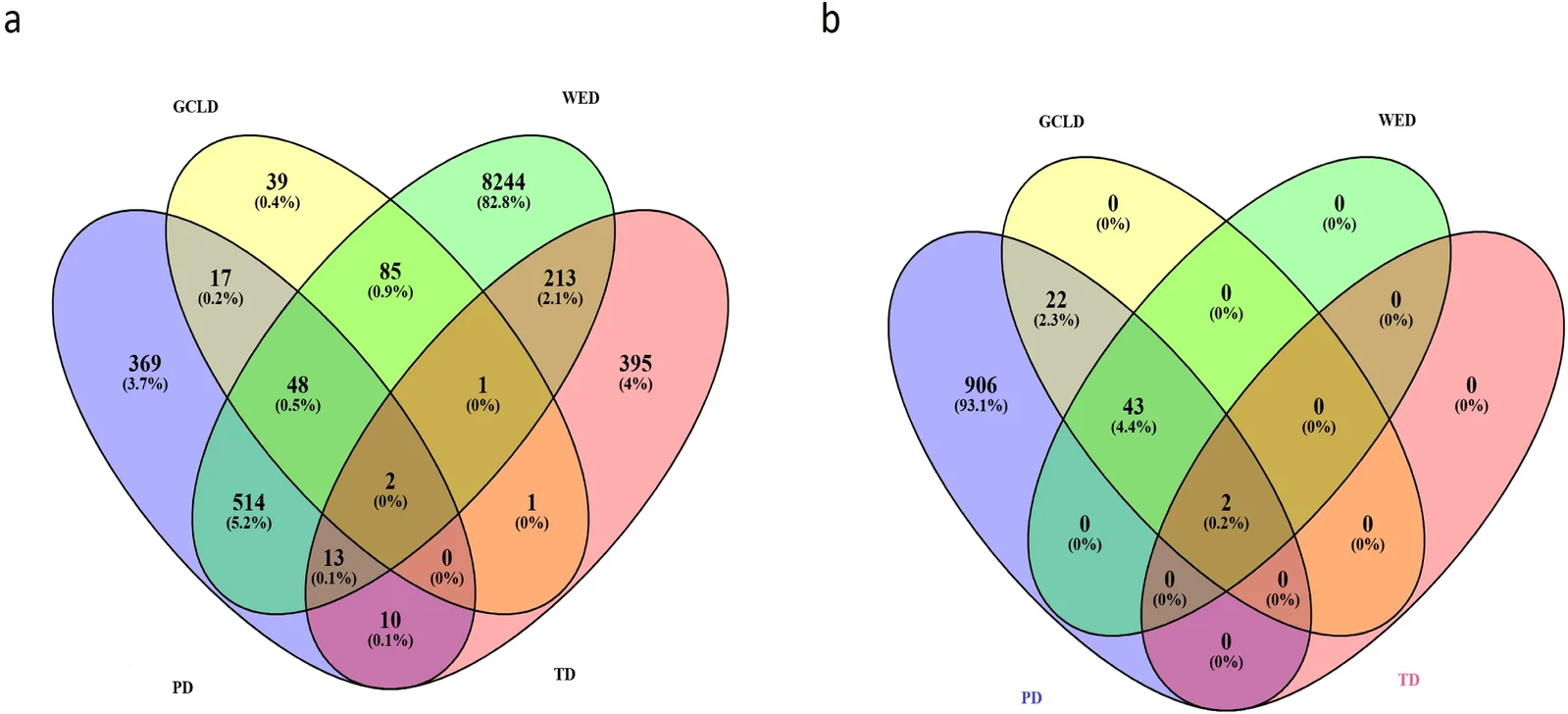
Venn diagram representing the overlap of driver genes across four datasets namely global CRC landscape driver genes (GCLD), all whole-exome driver genes from our cohort (WED), previously reported CRC driver genes (PD), and differentially expressed transcriptomic genes (TD). a) Gene overlap across driver discovery datasets. b) Overlap of confirmed driver genes.

More broadly, 13 genes intersected all three datasets (Supplementary Table 17), representing drivers that are validated globally, mutated in our cohort, and transcriptionally dysregulated. Conversely, the 2.5% overlap (25 genes) between PD and TD suggests functional impact through transcriptional disruption (Fig. 3a).

Conversely, the large unique components in PD and GCLD highlight that many globally identified drivers are absent in our specific cohort. As shown in the Venn diagram (Fig. 3b) genes are annotated as definitive drivers ('driver status = yes') within each dataset.

Our results mirror broader landscape studies that replicated only ∼5% of legacy drivers. This limited reproducibility stems from refined mutation models, aligning our data with the field’s shift toward more conservative, biologically grounded driver catalogues.

### Driver genes and other genes with second hits

The global landscape identified 56 driver genes with high biallelic inactivation and data from our cohort matched only 18 genes of which only four genes *ARID1A* (100%), *APC* (50%), *KMT2C* (50%), and *TP53* (16%) exhibited second hits (Supplementary Table 18). Among the remaining 137 global drivers, 53 genes in our cohort harboured biallelic variants, notably *FAT4, MYH11,* and *POLE* (Supplementary Tables 19, 20). Additionally, of the 306 genes ranked by somatic variant frequency, the top 10 with the highest biallelic prevalence are summarized in supplementary Table 21.

### Arm-level copy number alterations in colorectal cancer

GISTIC analysis of the entire cohort (n = 20) identified twelve chromosome arms with significant copy-number gains and 7 arms with significant losses (q < 0.25; Fig. 4a, Supplementary Table 22). The most frequent and statistically robust gains involved 19p (65%, q = 1.58 × 10⁻⁸), 19q (50%, q = 4.02 × 10⁻⁵), 16p & 16q (50% each, q = 6.5 × 10⁻⁴ and 5.7 × 10⁻⁴), and 17p (40%, q = 4.0 × 10⁻⁴), with additional significant gains at 17q, 20p, 20q, 22q, 7p, 7q and 8q. The most significant losses involved 21p (85%, q = 2.7 × 10⁻¹²) and 4q (65%, q = 3.2 × 10⁻⁸), with further significant deletions at 18q, 4p, 17p, 8p and 18p. Losses of 4q and 21p were near-ubiquitous across the cohort and present in both outcome groups (4q: 60% survivors, 70% non-survivors; 21p: 90% survivors, 80% non-survivors), suggesting these are early, outcome-independent events.

**Fig. 4:**
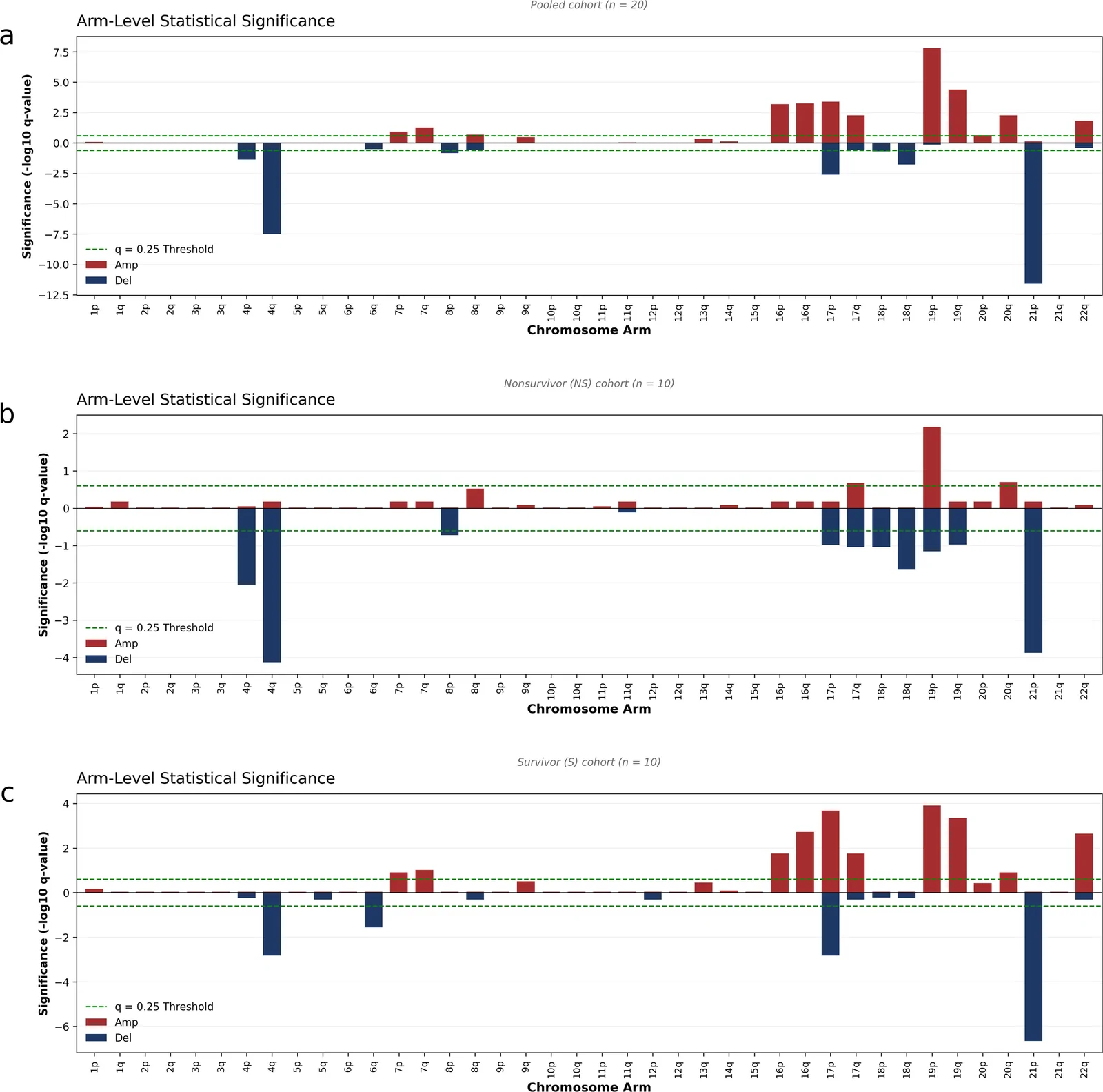
**Arm-level CNAs:** Gain and loss, and their significance. Arm-level CNA gain or loss across chromosomes. a) Significant Arm level CNAs in our cohort. b) Significant Arm level CNAs in non-survivors. c) Significant Arm level CNAs in survivors.

Stratification by outcome revealed distinct arm-level profiles (Fig. 4b-c). The non-survivor (NS) cohort (n = 10, Fig. 4b, Supplementary Table 23) showed a deletion-predominant profile: only 3 arms reached significance for gain (17q, 19p, 20q), against ten significant losses spanning 4p, 4q, 8p, 17p, 17q, 18p, 18q, 19p, 19q and 21p. Losses at 19p and 19q were exclusive to non-survivors, as were deletions at 4p, 8p, 17q and 18p and none of these reached significance in the survivor group (Fig. 4c, Supplementary Table 24).

In contrast, the survivor (S) cohort (n = 10, Fig. 4b, Supplementary Table 24) showed a gain-predominant profile, with ten significant amplifications (7p, 7q, 16p, 16q, 17p, 17q, 19p, 19q, 20q, 22q) and only four significant deletions (4q, 6q, 17p, 21p). Gains at 7p, 7q, 16p, 16q, 19q and 22q were unique to survivors, while loss of 6q was the only deletion specific to this group. Both outcome groups shared significant gain of 19p and 17q, and significant loss at 4q, 17p and 21p — 17p notably showed concordant gain and loss signals in both groups indicating a core set of shared arm-level events superimposed on divergent, outcome-specific patterns of predominant deletion in non-survivors versus predominant amplification in survivors.

### Comparison of Focal copy number gains and deletions

GISTIC analysis of our cohort (n = 20) identified 14 significantly amplified and 7 significantly deleted focal peaks (q < 0.25; Fig. 5a, Supplementary Table 25). Among the 14 significantly amplified focal peaks, several combined both high statistical significance and high frequency of alterations. 19q13.2 (AKT2 locus**)** was both highly significant and highly recurrent (q = 9.01 × 10⁻², 65%), as was 22q11.21, the single most significant amplification peak overall (q = 5.25 × 10⁻², 50%). 16p13.3 and 20q13.12 were the most frequently altered amplification peaks in the cohort (70% and 65%, respectively), though at comparatively lower significance (q = 1.51 × 10⁻¹ for both). 5p15.33 was notable for its high significance despite a lower alteration frequency (q = 1.23 × 10⁻¹, 20%). The remaining amplification peaks 8q24.3 (q = 1.51 × 10⁻¹, 45%), 9q34.3 (q = 1.51 × 10⁻¹, 40%), 17q21.31 (q = 1.99 × 10⁻¹, 45%), 6p21.1 (q = 1.51 × 10⁻¹, 35%), 12p13.31 (q = 1.51 × 10⁻¹, 35%), 11q13.1 (q = 1.51 × 10⁻¹, 30%), 1p21.1 (q = 1.51 × 10⁻¹, 25%), 15q26.1 (q = 2.20 × 10⁻¹, 40%), and 6q13 (q = 2.17 × 10⁻¹, 20%) showed lower combined significance and/or frequency.

**Fig. 5:**
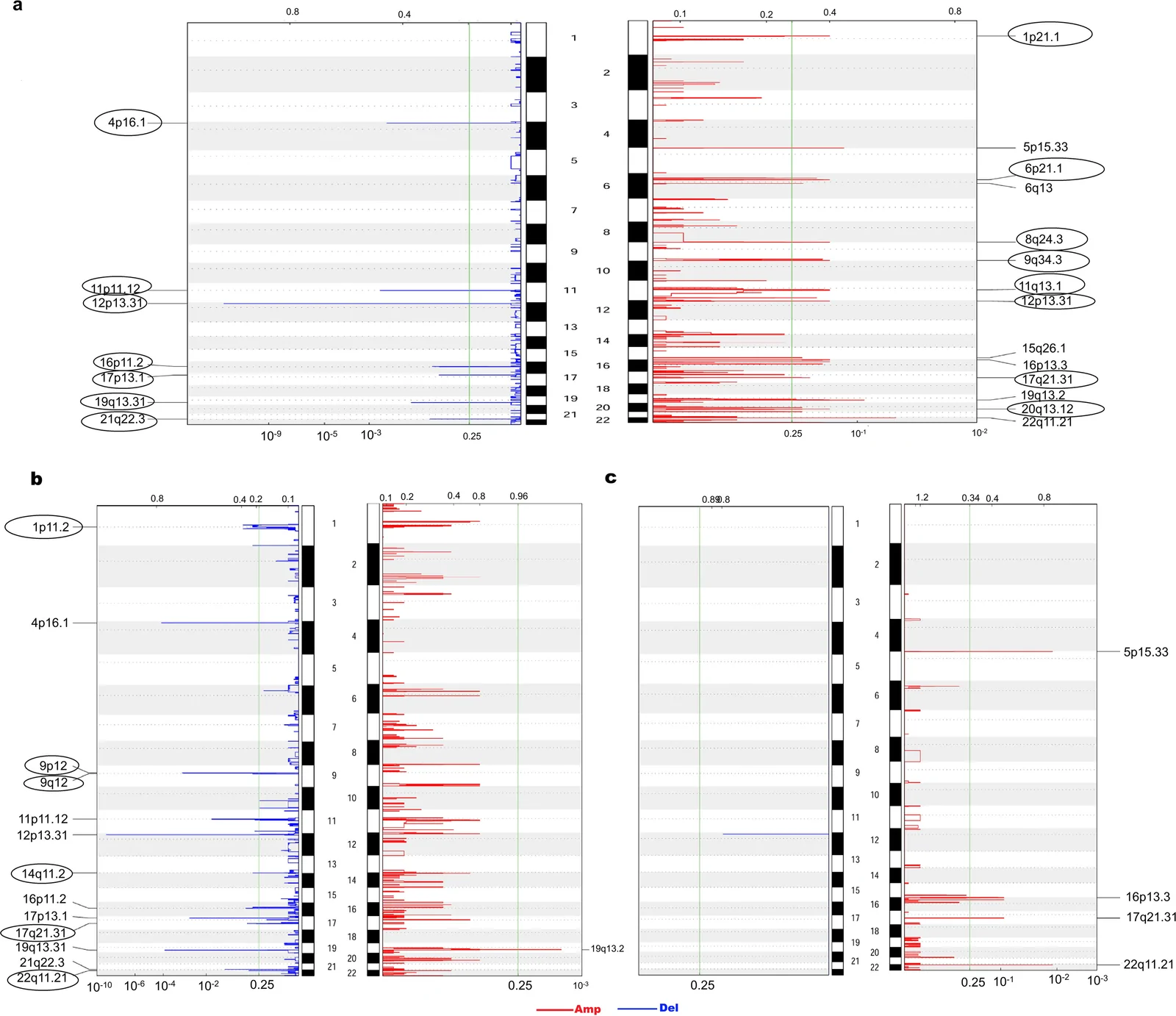
**Focal Copy Number (FCN) Analysis.** (a) Landscape of focal gains and deletions across the entire cohort. (b–c) Comparative analysis of focal genomic alterations in (b) non-survivors versus (c) survivors. Circled cytobands highlight exclusive copy number variations that distinguish the two groups.

Among the 7 significantly deleted focal peaks, 12p13.31 stood out as combining both the highest significance and a high frequency of alteration by a wide margin (q = 8.81 × 10⁻¹⁵, 45%), making it the single most robust deletion peak in the cohort on both metrics simultaneously. 4p16.1 also combined strong significance with a substantial alteration frequency (q = 4.00 × 10⁻³, 40%). 17p13.1 was the most frequently deleted peak overall (55%), though at comparatively lower significance (q = 7.82 × 10⁻²). 11p11.12 was highly significant (q = 2.45 × 10⁻³) but was altered at a markedly lower frequency (15%).

The remaining deletion peaks 21q22.3 (q = 5.13 × 10⁻², 40%), 19q13.31 (q = 1.90 × 10⁻², 35%), and 16p11.2 (q = 5.72 × 10⁻², 35%) showed intermediate significance and frequency values.

When compared against the global cohort, several focal peaks were only observed in our cohort (indicated by circled labels in Fig. 5a). All seven deletion peaks 4p16.1, 11p11.12, 12p13.31, 16p11.2, 17p13.1, 19q13.31, and 21q22.3 were observed only in our cohort. Among the amplification peaks, eight of the fourteen were observed only in our cohort: 1p21.1, 6p21.1, 8q24.3, 9q34.3, 11q13.1, 12p13.31, 17q21.31, and 20q13.12. The remaining six amplification peaks (5p15.33, 6q13, 15q26.1, 16p13.3, 19q13.2, and 22q11.21) overlapped with peaks that were also detected in the global cohort.

Stratification by outcome revealed a striking asymmetry (Fig. 5b-c), Supplementary Tables 26–27). Non-survivors were characterised almost entirely by focal losses: 13 deletion peaks reached significance, most prominently 12p13.31 (q = 1.39 × 10⁻⁹, 60%), 4p16.1 (q = 7.75 × 10⁻⁵, 50%), 19q13.31 (q = 1.22 × 10⁻⁴, 50%), 9p12 (q = 1.14 × 10⁻3, 40%) and 17p13.1 (q = 2.36 × 10⁻⁵, 70%), and 11p11.12, 21q22.3, 1p11.2, 16p11.2, 17q21.31, 22q11.21, 9q12 and 14q11.2. Only one gain, 19q13.2/AKT2 (q = 0.138, 50%) reached significance in this group.

Of the 13 non-survivor deletion peaks, six were observed only in this subgroup relative to our cohort and global data (Fig. 5b): 1p11.2, 9p12, 9q12, 14q11.2, 17q21.31, and 22q11.21.

Survivors showed an inverse pattern (Fig. 5c, Supplementary Tables 27): four amplifications reached significance 5p15.33 (q = 1.24 × 10^⁻2^, 30%), 22q11.21 (q = 1.24 × 10^⁻2^, 70%), 16p13.3 (q = 8.94 × 10^⁻2^, 80%) and 17q21.31 (q = 8.94 × 10^⁻2^, 50%) and with no significant deletions. Of these four gains, three were present in the overall cohort; only one gain peak at 22q11.21 observed in global data, which is not present in our cohort.

Overall, the focal-level results mirror the arm-level findings: non-survivors carry a burden of significant deletions with essentially no significant gains, whereas survivors show the reverse, having recurrent gains (notably 16p13.3, 22q11.21, 17q21.31) with no significant loss.

### Genetic features with an approved therapy

None of the tumors in our cohort harboured alterations linked to tumour-type–specific FDA/EMA-approved therapies, such as *BRAF* V600E, and all tumours were microsatellite stable (MSS). However, 9 tumors (45%) exhibited high tumour mutational burden (TMB-H; Supplementary Table 28) representing a feature of direct therapeutic relevance that needs additional validation.

However, 9 tumors (45%) were categorized as TMB-H (Supplementary Table 28). While TMB-high status may have potential clinical relevance in treatment decision-making within appropriate clinical settings, treatment eligibility and therapeutic benefit cannot be determined from our cohort in the absence of immunotherapy-related treatment-response data. These findings represent descriptive genomic biomarker measurements and require further prospective validation in larger cohorts.

### Genetic features with compelling biological evidence supporting therapeutic response

Two tumors in our cohort harboured somatic variants in the *ARID1A* gene, a biomarker associated with enhanced sensitivity to immune checkpoint inhibitors and *ATR* inhibitors in multiple tumour types. Additionally, *ERBB2* (HER2) amplification was identified in one tumour, and FGFR pathway alterations including *FGFR1* and *FGFR2* somatic variants were found in one tumour each. Mutations in *KRAS* (2 tumors), *NF1* (2 tumors), and *CDKN2A* (1 tumor) were also detected. No tumors harboured variants in *MTOR, FGFR3*, or *PTEN* (Supplementary Table 29).

### Approved and high-confidence drug targets

Several drivers in our dataset mapped to approved drug targets from the global data, offering established pathways for therapeutic intervention. Specifically, 17 of the 142 identified driver genes correspond to targets with either tumour-type-specific or tumour-agnostic approvals (Supplementary Table 30). Although some associated agents are not currently indicated for CRC, these druggable drivers suggest potential for drug-repurposing or participation in genotype-matched basket trials.

### Structurally tractable driver genes

Of 209 globally identified structurally tractable driver genes, 142 driver genes in our cohort matched this set. A substantial 33.8% (48 genes) are classified as "3D-ligandable," offering high-value targets for small-molecule discovery.

While 17 genes (11.97%) have approved therapies, only *PIK3R1* and *PLK1* (1.40%) are currently "Investigational." Furthermore, seven drivers—*ACVR1B, ATM, CASP8, CREBBP, NCOR2, TBK1, and TGFBR2*—are supported by high-quality chemical probes (Supplementary Table 30). These probes facilitate functional perturbation in experimental models, aiding the mechanistic prioritization of these drivers.

## Integration of dependency, selectivity, and druggability

We integrated data generated in our cohort to the Global genomic landscape and identified drivers with high therapeutic potential. Wnt pathway nodes, specifically *CTNNB1*, demonstrated the strongest essentiality (efficacy - 1.652) alongside confirmed structural tractability. *PIK3R1* and *PLK1* emerged as high-dependency investigational targets, while *ERBB3, IDH1, MAP2K1, SRC,* and *TOP1* combine high essentiality with authorized inhibitors.

In contrast, tumour suppressors like *ARID1B, ARID2*, and *BIRC6* showed significant negative effectiveness but lacked direct druggability. This aligns with loss-of-function biology, where therapeutic strategies typically require synthetic lethal interactions rather than direct inhibition.

## Discussion

Comparing genomic data from a small, under-represented cohort to larger global datasets aids biomarker discovery and understanding disease aetiology. While shared variants highlight conserved pathways, unique variants reveal ethnicity-specific mechanisms and therapeutic targets. In this study, we compared our cohort's exome and transcriptome data with a global whole-genome landscape of 2,023 colorectal tumors [9].

Our analysis revealed a distinct mutational landscape. De novo SBS signatures matched COSMIC v3 references, but we identified DBS17 (unknown etiology) in our cohort. Furthermore, ID19 (85%) and ID7 (30%; MMRd etiology) emerged as cohort-specific. Notably, ID7 co-occurred with SBS5, SBS1, and ID2— a pattern absent globally. In contrast, global SBS93 was absent in our cohort.

While global profiles often feature SBS88 and ID18 (associated with *pks+ E. coli* and colibactin in proximal tumors) [19,20], our cohort displayed a distal colon profile enriched with SBS10a/10b (POLE deficit). This ultra-mutated phenotype is typically associated with favourable prognosis and high immunogenicity. Although signatures were largely independent of survival status, SBS10a/b, SBS15, ID2, and ID7 showed trends toward better survival, and this warrants further investigation in larger cohorts.

The presence of SBS15 and ID7 signatures in pathologically confirmed MSS tumors indicates a decoupling between genome-wide substitution patterns and localized microsatellite slippage. This discordance likely reflects subtle MMR impairment or alternative hypermutation pathways [21] that do not meet the threshold for a classical MSI phenotype. Furthermore, the low activity of these signatures suggests a cumulative genomic footprint rather than acute repair failure.

In the global dataset, 193 driver genes were reported and our cohort shared 133 of these, showing high concordance with large-scale studies. However, core driver frequencies were significantly lower than global rates: *APC* (25% vs. 74%), *TP53* (30% vs. 67%), *KRAS* (10% vs. 40%), and *PIK3CA* (5% vs. 20%). This reiterates the limited prevalence of standard pathways and suggests alternate ethnicity-specific mechanisms [13]. Beyond, standard RAS drivers, we identified minor MAPK modifiers (*MAP2K1, MAP2K7, MAP3K21, RGS12*) [22], which likely serve as cooperative events and immune-escape drivers (*TAP1/2, HLA-A/B/C*) indicating distinct selective pressure on antigen presentation, a feature common to both MSI and a subset of MSS colorectal cancers.

To further assess the functional relevance of genomic drivers, we compared our dysregulated transcriptome data with global drivers that identified only three genes *MET, SOX9*, and *TGIF1* showing concordant somatic alterations and transcriptional dysregulation. These genes play critical roles in RTK signalling (*MET*), WNT modulation (*SOX9*), and TGFβ regulation (*TGIF1*). This limited overlap underscores CRC complexity, where most driver mutations exert effects through mechanisms not reflected at the steady-state mRNA level.

While global driver enrichment between survivors and non-survivors wasn't significant, gene-specific trends emerged. *APC* and *PRDM2* variants were more prevalent in non-survivors (40% vs. 10%), suggesting aggressive biology. Conversely, *TP53* and *PDE4DIP* variants were frequent in survivors (40% vs. 10%). These results suggest specific mutations reflect differential selective pressures, even if total driver burden doesn't predict survival.

Across the cohort, somatic variants were identified in 9,120 genes, of which 435 genes harboured recurrent damaging variants (≥2 occurrences; and predicted as deleterious by both PolyPhen-2/SIFT). The top 10 genes (*NLRC5, PRDM2, TP53, APC, HLA-A, PDE4DIP, RNF43, SETD1B, TCF7L2, ACVR2A* and *ANKRD11*) with the highest somatic variant burden across tumour samples are primarily involved in immune evasion and tumour suppression, aligning with selective pressures noted in global CRC studies.

Recurrent driver mutations signify positive selection. Compared to the ∼59 global recurrent CRC drivers, only Six drivers (*ACVR2A, BAX, PRDM2, RGS12, RNF43, TCF7L2*) exhibited cohort-specific recurrence not reported globally*. ACVR2A* and *TCF7L2* variants were enriched in survivors. Overall, 17 recurrent variants out of 48 recurrent variants were exclusively enriched in non-survivors, highlighting roles in disease aggressiveness.

The Indian CRC cohort displays a specific pattern of tumour suppressor loss via biallelic inactivation. While *APC, ARID1A*, and *KMT2C* showed high second-hit frequencies (50-100%), TP53 exhibited a second hit in only 16.7%. *ARID1A* inactivation was exclusive to non-survivors. This suggests mono-allelic TP53 mutations provide sufficient selective advantage, likely through dominant-negative effects.

When extending our evaluation to genomic alterations with strong biological or preclinical evidence for predicting sensitivity or resistance to targeted therapies, additional features emerged. Across the cohort, *ARID1A* mutations (n = 2), a biomarker associated with sensitivity to immune checkpoint inhibitors and *ATR* inhibitors across tumour types. *ERBB2* (*HER2*) amplification (n = 1), a potentially targetable alteration with anti-HER2 regimens (trastuzumab-based combinations) in metastatic CRC, supported by emerging clinical evidence. FGFR pathway alterations, including FGFR1 (n = 1) and FGFR2 (n = 1) mutations. Although not established targets in CRC, FGFR aberrations have shown therapeutic responsiveness in other cancers and may represent candidates for basket trials. *KRAS* mutations (n = 2), which primarily indicate resistance to anti- *EGFR*-targeted monoclonal antibodies were identified and retain strong predictive value for refractoriness to anti-*EGFR* therapy. *NF1* mutations (n = 2) may activate MAPK signalling and are increasingly recognised as potential biomarkers of MEK-inhibitor sensitivity in preclinical models. *CDKN2A* mutation (n = 1), suggests with dysregulated cell-cycle control and potential vulnerability to CDK4/6 inhibition in selected contexts. TMB-H again represented the most frequent actionable feature (n = 9) across this evidence tier. No samples showed *MTOR, FGFR3* or *PTEN* mutations within this group.

Beyond these canonical drivers, the cohort demonstrated consistent biallelic disruption in *FAT4* and *LAMA5* (both 100%), and recurrent loss in *TTN, HELZ2,* and *OBSCN*. Somatic variants in *MYH11, TTN, OBSCN, PAPPA2* and *HELZ2* were survival enriched, suggesting role in tumour establishment but do not independently dictate the reduction in patient survival time. Whereas variants in *CEP350, SLC8A3* and *LAMA5* were non-survival-enriched, identifying these as progression drivers that likely accelerate mortality. The lack of second hits in well-known genes like *MSH2* and *SMAD4* suggests alternate epigenetic silencing or haploinsufficiency, indicating a distinct driver architecture.

The focal-level GISTIC results provide gene-resolution support for the arm-level instability patterns seen in this cohort and align with established colorectal cancer (CRC) biology. The most robust deletion peak, 12p13.31, and the recurrent loss at 17p13.1, a region containing *TP53*, are consistent with the well-documented role of *TP53* pathway disruption and chromosome 17p loss in CRC progression and adverse prognosis. Similarly, 4p16.1 loss and 19q13.31 loss have both been implicated in more aggressive, chromosomally unstable CRC subtypes, reinforcing their emergence as markers enriched in non-survivors and need validation in a larger cohort. On the amplification side, 19q13.2 (encompassing *AKT2*) stood out as both highly significant and highly recurrent, and was the only gain peak significant in non-survivors, consistent with *AKT2*/PI3K-AKT pathway activation as a known driver of proliferative and invasive behaviour in CRC, rather than a purely favourable-prognosis event. By contrast, the amplification peaks enriched in survivors (16p13.3, 22q11.21, and 17q21.31) are less classically defined as CRC drivers, raising the possibility that these represent either passenger events accompanying a less genomically disrupted tumour genome, or an outcome-associated gains not yet well characterised in CRC and their prognostic relevance will require further evaluation.

Our cohort demonstrates a restricted spectrum of conventional actionable genomic alterations. Notably, the absence of microsatellite instability (MSI), *BRAF* V600E, and *NTRK1/3* fusions indicates a predominance of microsatellite-stable (MSS) disease and limited applicability of established CRC-specific targeted therapies. Despite MSI negativity, 45% of tumours were classified as tumour mutational burden–high (TMB-H), highlighting a distinct molecular profile. In this MSS-dominant cohort, TMB-H emerges as the principal therapeutic biomarker, supporting eligibility for immune checkpoint inhibitors under tissue-agnostic approvals. Overall, immunotherapy-related biomarkers appear to hold greater clinical relevance in this cohort than canonical CRC-targeted alterations need further validation. Additional variants in *KRAS, ARID1A*, and *NF1* were detected, though at lower frequencies compared with global reports.

Integration of functional dependency and druggability analyses indicates that non-druggable tumour suppressors dominate the therapeutic landscape, yet substantial clinical opportunities remain. Nearly half of the identified drivers (47.9%) lack pharmacological annotations, consistent with global CRC datasets characterised by prevalent loss-of-function alterations. However, 12.0% of drivers correspond to targets of licensed therapies, and 33.8% are structurally “3D-ligandable,” highlighting potential for future chemical probe development or synthetic lethal strategies. Drug repurposing and genotype-matched trial enrolment are supported by the presence of these licensed therapies, even if many of them are not standard for CRC. Comparative analysis with global initiatives further distinguishes non-actionable foundational mutations from a subset of therapeutically tractable alterations, offering a framework to guide precision oncology strategies in this cohort.

This study has several limitations. The modest cohort size, tumor-only design, absence of patient-matched normal samples, small panel of normals, and possibility of residual rare germline variants represent limitations of the somatic variant analysis. Accordingly, the genomic findings are presented as candidate/hypothesis-generating observations requiring validation in independently collected cohorts and, where possible, matched-tumor-normal sequencing. Because sample size constraints exclude multivariable Cox regression to control for stage, these data cannot support claims that specific somatic variants or pathways directly accelerate disease progression or mortality. Functional validation of putative population-specific drivers and mutational signatures was beyond the scope of this work and requires confirmation in independent cohorts and experimental models. Furthermore, the lack of prospective treatment and response data precluded direct evaluation of therapeutic predictive value, underscoring the need for larger, longitudinal studies in ethnically diverse populations.

## Conclusion

In conclusion, integrated analysis of mutational signatures, driver architecture, transcriptomic dysregulation, and druggability reveals a colorectal cancer landscape shaped by evolutionary and therapeutic constraints not fully represented in global datasets. The distinct driver and mutational signature patterns observed in this cohort warrant validation in larger studies and provide a framework for developing population-tailored biomarkers and precision therapeutic strategies.

## Funding

No Applicable Funding.

## Ethics approval and consent to participate

The study was conducted in accordance with the Declaration of Helsinki and was approved by the Institutional Ethics Committee of the Asian Institute of Gastroenterology (Approval No: AIG/IEC34/07.2019-19). Written informed consent was obtained from all patients before their inclusion in the study and for the collection of tumor and adjacent normal tissues for genomic and transcriptomic profiling.

## CRediT authorship contribution statement

**Anil Vashapaka:** Writing – original draft, Formal analysis. **Ravikanth Vishnubhotla:** Writing – review & editing, Supervision, Conceptualization, Investigation, Project administration. **Rao GV:** Methodology, Resources, Writing – review & editing. **Pradeep Rebala:** Methodology, Writing – review & editing. **Sanjeev M Patil:** Methodology, Writing – review & editing. **Mahesh Shetty:** Methodology, Writing – review & editing. **K Srinivas Naik:** Writing – review & editing. **Anuradha Sekaran:** Methodology. **Nageshwar Reddy D:** Methodology, Resources, Writing – review & editing.

## Declaration of competing interest

The authors declare that they have no known competing financial interests or personal relationships that could have appeared to influence the work reported in this paper.

## Data Availability

The raw whole-exome and transcriptome sequencing data generated in this study have been deposited in the Indian Nucleotide Data Archive (INDA) of the Indian Biological Data Centre (IBDC) under accession number INRP000775, and are also available through the International Nucleotide Sequence Database Collaboration (INSDC) under BioProject accession PRJEB128235. Processed data supporting the findings of this study are available within the article and its Supplementary Materials.

## References

[1] Matsuda T., Fujimoto A., Igarashi Y. (2025). Colorectal cancer: epidemiology, risk factors, and public health strategies. Digestion.

[2] Sung H., Ferlay J., Siegel R.L., Laversanne M., Soerjomataram I., Jemal A. (2021). Global cancer statistics 2020: GLOBOCAN estimates of incidence and mortality worldwide for 36 cancers in 185 countries. CA Cancer J. Clin..

[3] Xi Y., Xu P. (2021). Global colorectal cancer burden in 2020 and projections to 2040. Transl. Oncol..

[4] Morgan E., Arnold M., Gini A., Lorenzoni V., Cabasag C.J., Laversanne M. (2023). Global burden of colorectal cancer in 2020 and 2040: incidence and mortality estimates from GLOBOCAN. Gut.

[5] Roshandel G., Ghasemi-Kebria F., Malekzadeh R. (2024). Colorectal cancer: epidemiology, risk factors, and prevention. Cancers.

[6] Shivshankar S., Patil P.S., Deodhar K., Shrikhande S.V., Sirohi B., Gulia A. (2025). Epidemiology of colorectal cancer: a review with special emphasis on India. Indian J. Gastroenterol..

[7] Raj S., Kishor K., Devi S., Singh N.K., Kumar A., Bharti A.C. (2024). Epidemiological trends of colorectal cancer cases in young population of Eastern India: a retrospective observational study. J. Can. Res. Ther..

[8] Agrawal M., Surendran A.K., Venkatesh K.K., Sharma A., Pandey D., Misra S. (2025). Screening for colorectal carcinoma in India: real-world scenario, pitfalls, and solutions. South Asian J. Cancer.

[9] Cornish A.J., Gruber A.J., Kinnersley B., Knight S.J., Engebretsen S., Seneviratne A.K. (2024). The genomic landscape of 2,023 colorectal cancers. Nature.

[10] Spratt D.E., Chan T., Waldron L., Speers C., Feng F.Y., Pierce L.J. (2016). Racial/ethnic disparities in genomic sequencing. JAMa Oncol..

[11] Andrade C. (2020). Sample size and its importance in research. J. Clin. Psychiatry.

[12] Lehmann B., Bräuninger L., Cho Y., Kim K., Park S., Jones A. (2025). Methodological opportunities in genomic data analysis to advance health equity. Nat. Rev. Genet..

[13] Vishnubhotla R., Avanthi S., Mitnala S., Rao P.N., Reddy D.N., Bharti A.C. (2024). Lesser prevalence of polyps/WNT-dysregulation and concomitant upregulation of gamma-catenin/MYC point to alternate pathways in colorectal cancer in India. Mol. Carcinog..

[14] Alexandrov L.B., Nik-Zainal S., Wedge D.C., Aparicio S.A., Behjati S., Biankin A.V. (2013). Signatures of mutational processes in human cancer. Nature.

[15] Alexandrov L.B., Kim J., Haradhvala N.J., Huang M.N., Tian Ng A.W., Wu Y. (2020). The repertoire of mutational signatures in human cancer. Nature.

[16] Talevich E., Shain A.H., Botton T., Bastian B.C. (2016). CNVkit: genome-wide copy number detection and visualization from targeted DNA sequencing. PLoS. Comput. Biol..

[17] Mermel C.H., Schumacher S.E., Hill B., Meyerson M.L., Beroukhim R., Getz G. (2011). GISTIC2.0 facilitates sensitive and confident localization of the targets of focal somatic copy-number alteration in human cancers. Genome Biol..

[18] Avanthi S.U., Vishnubhotla R., Sasikala M., Mitnala S., Rao P.N., Reddy D.N. (2025). A gene panel to predict response to adjuvant chemotherapy and risk of recurrence in colorectal cancer. Malignancy Spectrum.

[19] Dziubańska-Kusibab P.J., Berger H., Battistini F., Iturbide M., Friedland N., Lamy R. (2020). Colibactin DNA-damage signature indicates mutational impact in colorectal cancer. Nat. Med..

[20] Chen B., Ramazzotti D., Heide T., Curtis C., Caravagna G. (2023). Contribution of pks+ E. coli mutations to colorectal carcinogenesis. Nat. Commun..

[21] Campbell B.B., Light N., Fabrizio D., Zatzman M., Fuligni F., de Borja R. (2017). Comprehensive analysis of hypermutation in human cancer. Cell.

[22] Guo Y.J., Pan W.W., Liu S.B., Shen Z.F., Xu Y., Hu L.L. (2020). ERK/MAPK signalling pathway and tumorigenesis. Exp. Ther. Med..

