# Supplementary material for: Integrated genomic and transcriptomic profiling reveals distinct molecular features of colorectal cancer in an Indian cohort
Source: Neoplasia. 2026 Oct 16;83:101381. doi: 10.1016/j.neo.2026.101381 (PMC13738135; doi:10.1016/j.neo.2026.101381)
Supplement: Supplementary file 1 [file mmc1.docx]

**Integrated Genomic and Transcriptomic Profiling Reveals Distinct Molecular Features of Colorectal Cancer in an Indian Cohort**

Anil Vashapakaª, Ravikanth Vishnubhotlaª^,*^, Rao GVᵇ, Pradeep Rebalaᵇ, Sanjeev M Patilᵇ, Mahesh Shettyᵇ, K Srinivas Naik^c^, Anuradha Sekaran^b^, Nageshwar Reddy D^b^.

^a^ Asian Healthcare Foundation, Plot No 2/3/4/5, Survey No 136/1, Mindspace Road, Gachibowli, Hyderabad, Telangana 500032, India.

^b^ AIG Hospitals Plot No 2/3/4/5, Survey No 136/1, Mindspace Road, Gachibowli, Hyderabad, Telangana 500032, India.

^c^ Associate Professor, Centre for Plant Molecular Biology, Osmania University, Tarnaka, Hyderabad – 500007, Telangana

***Correspondence to:**

Ravikanth Vishnubhotla,

Asian Healthcare Foundation, a research wing of AIG Hospitals,

Mindspace Road, Gachibowli, Hyderabad- 500032,

Telangana, India.

Email – [ravikanth@ahcf.co.in](mailto:ravikanth@ahcf.co.in); ravikanth.aig@gmail.com

**Telephone**: +91-40-42444222; **Fax**: +91-40-23324255

**Supplementary Figures Captions**

**Supplementary Figure 1.** Somatic variant calling, annotation, and RNA-Seq pipelines. a) Whole exome sequencing workflow, b) Transcriptome sequence workflow, c) Copy number alteration workflow, d) Cosmic mutational signature workflow.

**Supplementary figure 2.** Total 133 Driver gene mutation distribution

**Supplementary Figure 3**: Distribution of pathogenic variants per gene a) 26 pathogenic variants shared by 15 genes. b) Distribution of 175 variants in 159 genes and 1 c) Cohort-specific distribution of pathogenic variants in 3840 genes.

**Supplementary Methods**

***Tissue Collection***

In this prospective study colorectal tumour tissues (n=20) and non-malignant colorectal control tissues (n=5) were collected from patients undergoing surgical resection for colorectal cancer (CRC) and non-oncological indications respectively. The study was conducted between May 2013 and December 2017. All samples were immediately preserved in RNAlater (Thermo Fisher Scientific) to maintain nucleic acid integrity. Clinical follow-up was maintained for a minimum of 36 months for all participants. The patients classified as Survivors were followed-up for a minimum of 36 months and the survivor status confirmed. Patients who expired anytime during the study period were classified as non-survivors. All the patients underwent surgery followed by chemotherapy (adjuvant).

**Stage/Nodal Confounding and Power Limitations:** Subgroup stratification into survivors (n=10) and non-survivors (n=10) is constrained by statistical power. Furthermore, baseline clinical characteristics demonstrate a substantial imbalance in nodal status (N-stage) between cohorts (Supplementary Table ST31), with non-survivors enriched for nodal metastasis (N1–N2) and advanced stage. As multivariable statistical adjustment (e.g., Cox regression) is unfeasible with N=20, observed mutational differences between outcome groups are strongly confounded by underlying stage heterogeneity and should not be interpreted as independent prognostic drivers

***Ethical Considerations***

The study protocol was approved by the Institutional Research and Ethics Committee of the Asian Institute of Gastroenterology (AIG/IEC34/07.2019-19) and adhered to the ethical principles of the 1975 Declaration of Helsinki. Written informed consent was obtained from all participants before enrolment.

***DNA Extraction and Whole Exome Sequencing (WES)***

Genomic DNA was isolated from tissue samples using the QIAamp DNA Kit (Qiagen, Hilden, Germany) according to the manufacturer’s instructions. WES libraries were constructed using the Twist Human Customized Core Exome Kit. Sequencing was performed on the Illumina HiSeq platform (Illumina, San Diego, CA) utilizing a 2×150 bp paired-end configuration. To ensure high-resolution variant detection, a minimum mean target coverage of 500X was achieved across all samples.

***Somatic Variant calling from WES:***

1. Data Quality Control and Read Alignment

Raw sequencing reads in FASTQ format were first evaluated for quality using FastQC v0.12.1 [1] To ensure high-quality mapping, adapter sequences and low-quality bases were trimmed where necessary. The processed reads were aligned to the human reference genome hg19_v0_Homo_sapiens_assembly19.fasta using the BWA-MEM2 v2.3 [2] algorithm, which utilizes SIMD instructions for accelerated alignment while maintaining identical results to the original BWA-MEM.

2. Alignment post-processing

The resulting alignments were processed using Samtools v1.22 [3] and Picard Tools v3.4.0 [4]. Coordinate sorting and indexing were performed via Samtools. To mitigate potential PCR artifacts, duplicate reads were identified and marked using Picard MarkDuplicates.

3. Base Quality Score Recalibration (BQSR)

To correct for systematic errors in base quality scores, we performed Base Quality Score Recalibration using GATK4 (v4.5) [5]. We first generated a recalibration table with BaseRecalibrator using known variant sites from dbSNP and the 1000 Genomes Project as a gold standard. These recalibration models were then applied to the data using ApplyBQSR, resulting in analysis-ready BAM files.

4. Somatic Variant Discovery

Somatic single-nucleotide variants (SNVs) and small insertions/deletions (indels) were identified using GATK4 Mutect2.

- Panel of Normals (PoN): To filter out recurrent sequencing artifacts and germline leakage, a cohort-specific Panel of Normals was constructed. This PoN was generated by processing the 5 non-malignant control samples through the Mutect2 "tumor-only" mode, followed by aggregation using CreateSomaticPanelOfNormals.
- Variant Calling: For each tumor sample, Mutect2 was executed utilizing the cohort-specific PoN and gnomAD v2.1.1 (exome) to further exclude population-level germline variants.

The use of a Panel of Normals (PoN) in place of matched normal samples is a robust and widely accepted strategy in somatic variant calling, as it effectively filters recurrent technical artefacts, sequencing noise, and population-level germline variants, thereby enhancing specificity and maintaining high confidence in true somatic variant detection, particularly in studies where matched normal tissue is unavailable or may introduce its own biases

5. Variant Filtering and Refinement

Raw somatic calls were refined using the FilterMutectCalls module. We integrated cross-sample contamination estimates and read-orientation bias metrics to minimize false positives. Final variant processing and formatting were performed using bcftools v1.20 [6], while bedtools v2.31 [7] was utilized to restrict analysis to the specific exome capture target regions.

**Variant annotation**

6. Variant Functional Annotation and Prioritization

High-confidence somatic variants were functionally annotated using ANNOVAR (version 2025Mar02) [8]. We integrated several databases to assess the biological impact and clinical relevance of each alteration:

- Gene-Based Annotation: Variants were mapped to the RefSeq (refGene) gene set (hg19) to identify their genomic locations (e.g., exonic, intronic, intergenic). For exonic variants, the specific amino acid changes and functional consequences (e.g., nonsynonymous SNVs, frameshift indels, and stop-gain) were determined.
- Population Frequency Filtering: To isolate rare somatic events, variants were cross-referenced against multiple population-scale datasets. We utilized the gnomAD v2.1.1 (exome and genome), 1000 Genomes Project (2015 Aug), and avsnp151 databases. Variants with an allele frequency > 0.01 (1%) in any of these global populations were excluded from further analysis as potential common polymorphisms.
- Clinical Significance and Pathogenicity: Clinical interpretation was performed using the ClinVar database (version 20250721). Variants were prioritized based on their clinical significance; those classified as "benign" or "likely benign" were omitted. Furthermore, any variant with a "germline" origin annotation in ClinVar was excluded to focus strictly on somatic alterations.

**Variant Prioritization:**

To ensure high-confidence variant calls in our cohort variants, we implemented a stringent filtering pipeline following exome annotation. Variants were retained only if they reached a minimum sequencing depth of 200x, with the variant allele fraction (VAF) representing at least 25% of the total reads. Clinical filtering was performed using ClinVar; variants annotated as 'benign' or 'likely benign,' as well as those of germline origin, were excluded. Finally, the analysis was restricted to non-synonymous and exonic alterations by filtering out all silent (synonymous) and intronic variants.

Applying a ≥25% variant allele frequency (VAF) threshold in the context of deep sequencing (~500× coverage) is a deliberately conservative but well-justified approach, as it prioritizes high-confidence somatic calls by minimizing false positives arising from sequencing errors, PCR artefacts, and low-level contamination; at this depth, true clonal or near-clonal variants are expected to be robustly represented, and the stringent cutoff ensures excellent specificity and reproducibility across samples, which is particularly important when using a Panel of Normals instead of matched controls. These criteria resulted in 16,440 variants before after prioritization from the resulted 22,485 variants before in-house prioritization.

**Tumor Mutational Burden (TMB):**

TMB was calculated as the total number of non-synonymous somatic single nucleotide variants (SNVs) and indels per megabase (Mb) across the coding capture territory (33.0 Mb); Twist Human Customized Core Exome). Analysis was restricted to exonic variants meeting stringent filter criteria (sequencing depth ≥200x, variant allele frequency [VAF] ≥25%). A threshold of 10 mutations/Mb was applied to define TMB-High (TMB-H) status according to FDA tissue-agnostic guidelines.

**Microsatellite Instability (MSI) Assessment**

Microsatellite instability (MSI) was assessed using immunohistochemistry (MLH1/MSH2/MSH6/PMS2) or next-generation sequencing of 76 markers depending on tissue availability. The tumours were classified to be Microsatellite Instable (MSI) when the tissue had loss of nuclear expression in either one or more markers of the panel whereas the tumours with no detectable alterations were designated as Microsatellite Stable (MSS). For MSI analysis on the NGS, MSI Call, a bioinformatics pipeline developed as a torrent suite plugin for analysis of BAM Files generated on the Ion GeneStudio S5 was used. This plugin grouped the reads mapped to the marker’s location and measured the homopolymer signal from each read. It had then evaluated the signal statistics across the reads and estimated an MSI Score for each marker. Further, basic noise filter was applied and the total MSI Score was obtained by adding the individual marker scores. On completion of the analysis, the software itself had auto called whether the sample was either a MSS or MSI based on the alteration observed against the control provided in the run.

**Mutational Signature Analysis**

To investigate the mutational processes operative in the CRC cohort, we performed mutational run independently using the analysis-ready Mutect2 VCF files before application of the downstream in-house prioritization criteria. Signature extraction and decomposition using SigProfilerExtractor (v1.1) [9].

1. De Novo Extraction and COSMIC Mapping

To characterize the operative mutational processes, single base substitutions (SBS), doublet base substitutions (DBS), and small insertions/deletions (IDs) were extracted *de novo* using SigProfilerExtractor (v1.1) [9]. The extraction process utilized random initialization with 500 NMF replicates, spanning 10,000 to 1,000,000 iterations to ensure mathematical convergence. We explored a signature range of 1 to 15 for SBS, 17 for DBS, and 1 to 19 for IDs, maintaining default settings for all other algorithmic parameters.

Extracted *de novo* signatures were cross-referenced and decomposed into the COSMIC (v3.2) reference database. To ensure high-fidelity mapping, somatic mutations were first assigned to their respective SBS96, DBS78, and ID83 contexts using SigProfilerMatrixGenerator [10].

2. Quantifying Mutational Activity

Once *de novo* signatures were identified, we utilized SigProfilerAssignment to decompose them into known biological processes. The extracted signatures were mapped against the COSMIC v3.x Reference Database.

- Refitting Algorithm: We applied a Non-negative Least Squares (NNLS) algorithm to calculate the contribution of each COSMIC signature to individual samples.
- CRC-Specific Processes: Special attention was given to signatures highly prevalent in colorectal cancer, including SBS1/SBS5 (clock-like/aging), SBS6/SBS15/SBS26/SBS44 (Mismatch Repair Deficiency), and SBS10a/b (Polymerase Epsilon mutations).
- Indel Dynamics: Indel signatures were mapped to processes such as ID2 (slippage during DNA replication) and ID7 (Defective DNA Mismatch Repair).

For downstream analysis and clinical correlation, mutational signatures were quantified using three distinct metrics:

Presence–Absence: A binary assessment used to determine the shared aetiology across the cohort.

Proportional Activity: Defined as the relative fraction of mutations attributed to a specific signature within a single tumor, facilitating intra-sample comparisons.

Absolute Mutational Burden: Estimated by multiplying the signature activity by the total mutation count (SBS, DBS, or ID) for that sample. This provided an approximation of the total mutation load contributed by a specific biological process (e.g., the number of mutations caused specifically by MMR deficiency).

**CNA Calling using CNVkit v0.9.12**

CNVkit [11], which calculates relative copy number from normalised read depth across targeted and off-target areas, was used to create genome-wide copy number profiles. Log2 copy number ratios were computed for each genomic bin after read counts from Twist Human Customized Core Exome kit baited, and flanking regions were combined and normalised against the reference panel.

Circular binary segmentation (CBS) was used for segmentation in order to find contiguous genomic areas with constant copy number states. After that, segment-level log2 ratios were scaled and centred to take sample-specific coverage variability into consideration. In line with previous large-scale cancer exome investigations, segmented copy number profiles for each tumour were transformed to absolute copy number states using approximated tumour ploidy assumptions.

Copy-neutral, gain, and loss events were categorized by applying thresholding to the segmented log2 ratios. To focus on high-confidence alterations, segments were further filtered based on their weight and the number of probes covered.

The main input for cohort-level recurrence analysis was CNVkit outputs (*.cnr and *.cns files).

**GISTIC2.0**.

To identify significantly recurrent somatic copy number alterations across the cohort, segmented copy number profiles derived from CNVkit were analyzed using GISTIC2.0. [12] GISTIC2.0 [12] identifies genomic regions that are gained or lost more frequently than expected by chance, accounting for both the amplitude and frequency of copy number changes while controlling for background genomic instability.

Segmented copy number data were converted into GISTIC-compatible input format, and analyses were performed using standard parameters optimized for exome-derived copy number profiles.

To identify genomic regions significantly enriched for amplifications and deletions across the cohort, we utilized GISTIC 2.0.23 [12]. The segmented data generated by CNVkit was formatted into a .seg file and processed using the following parameters:

- Thresholding and Significance: Amplification and deletion thresholds were set at 0.1 (ta and t), and the confidence level for identifying peaks was set to 0.99.
- Broad vs. Focal Alterations**:** The -broad 1 and -brlen 0.7 parameters were applied to categorize alterations as broad (affecting >70 of a chromosome arm) or focal. To ensure a refined analysis of focal events, the -armpeel 1 option was used to "peel off" the effects of arm-level changes.
- Gene-Level Scoring: We utilized the -genegistic 1 and -savegene 1 flags to calculate significance scores for individual genes. To account for high-level focal events, the "extreme" Gene Cutoff Method (-gcm extreme) was employed, with a maximum cap of 1.5 for log2 ratios.
- Reference and Mapping: All coordinates were mapped to the hg19 human reference genome assembly (hg19.mat).

Significance thresholds were defined using false discovery rate–corrected q-values, with regions achieving q < 0.25 considered significantly recurrent, consistent with prior TCGA and pan-cancer studies. Broad events were defined as alterations spanning more than 50% of a chromosome arm, whereas focal events were restricted to smaller genomic intervals.

**Arm-level copy number alterations**

Arm-level CNAs were identified based on the proportion of each chromosome arm affected by gain or loss across samples. Recurrent arm-level gains and deletions were interpreted as candidate large-scale driver events and compared with previously reported colorectal cancer CNA landscapes.

Arm-level copy number profiles were summarized as gain, loss, or neutral states per chromosome arm for each tumor and used for downstream comparative and subgroup analyses.

**Focal copy number alterations and candidate driver regions**

Focal copy number peaks identified by GISTIC2.0 [12] were mapped to minimal common regions of amplification or deletion. Genes residing within these significant focal peaks were considered candidate copy number–driven genes. Previously reported colorectal cancer driver loci (e.g., recurrent gains involving oncogenes and deletions encompassing tumor suppressor genes) were evaluated alongside novel focal regions emerging in the cohort.

Minimal common regions were defined as the smallest overlapping genomic intervals shared across tumors contributing to each significant GISTIC peak.

Reported percentages represent GISTIC broad-frequency proportions across callable territory. In the main text and supplementary tables, exact integer sample counts (k/N) are provided alongside GISTIC broad frequency estimates to ensure clarity (N=20 overall cohort; n=10 non-survivors; n=10 survivors).

**Quality control and downstream integration**

Samples exhibiting excessive noise, highly fragmented segmentation profiles, or implausible genome-wide copy number distributions were excluded from CNA-based downstream analyses. Only tumors with stable segmentation and interpretable copy number profiles were retained.

Recurrent CNAs identified through GISTIC2.0 [12] were compared with previously published colorectal cancer global cohorts

**RNA-Seq**

**1. RNA-Seq Quality Control and Alignment**

Raw sequence reads in FASTQ format were subjected to quality assessment using FastQC v0.12.1 [1] to evaluate base quality scores, adapter contamination, and GC content. The reads were aligned to the human reference genome hg19 (matching the WES reference, hg19_v0_Homo_sapiens_assembly19.fasta) to keep genomic coordinates consistent across WES and RNA-seq using HISAT2 [13], a fast and sensitive alignment program for mapping next-generation sequencing reads. HISAT2 [13] was specifically chosen for its ability to handle splice-aware alignments, which is critical for transcriptomic data.

**2. Gene Quantification**

Following alignment, the resulting SAM/BAM files were processed to quantify gene expression levels. We utilized featureCounts [14] (part of the Subread package), a highly efficient program for assigning sequence reads to genomic features. Reads were mapped to the GENCODE v19 (hg19-matched), consistent with the RefSeq/refGene annotation. Only uniquely mapped reads were considered for downstream expression analysis to ensure high specificity in quantification.

**3. Differential Expression Analysis**

Differential gene expression (DGE) analysis between tumor (n=20) and control (n=5) tissues was performed using the DESeq2 [15] package in the R statistical environment.

- Normalization**:** DESeq2 applied the median-of-ratios method to account for differences in sequencing depth and library composition between samples.
- Statistical Modelling**:** The tool utilized a generalized linear model (GLM) based on the negative binomial distribution to model the count data.
- Significance Thresholds: Genes were considered significantly differentially expressed if they met a threshold of an adjusted P-value (P_adj) < 0.05 (applying the Benjamini-Hochberg false discovery rate correction) and a log_2{Fold Change} | > 1 (or your specific threshold).
- DESeq2 results with a total of 4,189 dysregulated genes expressions when comparing tumour versus normal tissues
- Out of 4,189 total dysregulated genes, 814 genes were identified with significance defined as adjusted p-value < 0.05 (Benjamini-Hochberg correction) and |log2 fold change| ≥ 1. Of these, 569 upregulated and 245 downregulated genes (814 total) met the significance threshold.

**Batch effect**

To eliminate technical batch effects, all 25 samples (tumors and controls) were processed simultaneously using the exact same library preparation, sequencing technique, and bioinformatics workflow. Furthermore, exploratory principal component analysis (PCA) confirmed the absence of technical clustering prior to downstream modeling. Raw counts were then processed directly through DESeq2, which accounts for library size differences via its built-in normalization.

**Gene dependency and structural druggability metrics**—including functional efficacy scores (e.g., CTNNB1 efficacy score of −1.652) were not independently calculated; rather, they were extracted directly from the annotated reference global CRC landscape dataset [16] to contextualize the therapeutic potential of the driver genes identified in our cohort.

**References**

1. Andrews S. FastQC: a quality control tool for high throughput sequence data. Babraham Bioinformatics 2010.
2. Vasimuddin M, Misra S, Li H, Aluru S. Efficient architecture-aware acceleration of BWA-MEM for multicore systems. IEEE Int Parallel Distrib Process Symp (IPDPS) 2019;314-24.
3. Danecek P, Bonfield JK, Liddle J, Marshall J, Stack A, Li H, et al. Twelve years of SAMtools and BCFtools. GigaScience 2021;10:giab008.
4. Broad Institute. Picard toolkit. GitHub Repository 2019
5. Van der Auwera GA, O'Connor BD. Genomics in the cloud: using Docker, GATK, and WDL in Terra. O'Reilly Media; 2020.
6. Danecek P, Bonfield JK, Liddle J, Marshall J, Stack A, Li H, et al. Twelve years of SAMtools and BCFtools. GigaScience 2021;10:giab008.
7. Quinlan AR, Hall IM. BEDTools: a flexible suite of utilities for comparing genomic features. Bioinformatics 2010;26:841-2.
8. Wang K, Li M, Hakonarson H. ANNOVAR: functional annotation of genetic variants from high-throughput sequencing data. Nucl eic Acids Res 2010;38:e164.
9. Islam SMA, Díaz-Gay M, Wu Y, Barnes M, Bergstrom EN, He Y, et al. Uncovering novel mutational signatures by de novo extraction with SigProfilerExtractor. Cell Genom 2022;2:100179.
10. Bergstrom EN, Huang MN, Mahto U, Barnes M, Stratton MR, Rozen SG, et al. SigProfilerMatrixGenerator: a tool for visualizing and exploring patterns of small mutational events. BMC Genomics 2019;20:685.
11. Talevich E, Shain AH, Botton T, Bastian BC. CNVkit: genome-wide copy number detection and visualization from targeted DNA sequencing. PLoS Comput Biol 2016;12:e1004871.
12. Mermel CH, Schumacher SE, Hill B, Meyerson ML, Beroukhim R, Getz G. GISTIC2.0 facilitates sensitive and confident localization of the targets of focal somatic copy-number alteration. Genome Biol 2011;12:R41.
13. Kim D, Paggi JM, Park C, Bennett C, Salzberg SL. Graph-based genome alignment and genotyping with HISAT2 and HISAT-genotype. Nat Biotechnol 2019;37:907-15.
14. Liao Y, Smyth GK, Shi W. featureCounts: an efficient general purpose program for assigning sequence reads to genomic features. Bioinformatics 2014;30:923-30.
15. Love MI, Huber W, Anders S. Moderated estimation of fold change and dispersion for RNA-seq data with DESeq2. Genome Biol 2014;15:550.
16. Cornish AJ, Gruber AJ, Kinnersley B, Knight SJ, Engebretsen S, Seneviratne AK, et al. The genomic landscape of 2,023 colorectal cancers. Nature 2024;633:127-36.
